# Heterogeneous Medial Prefrontal Cortex Ensembles Exhibit Reproducible Internal Temporal Dynamics during Choice Behavior

**DOI:** 10.1523/JNEUROSCI.0358-26.2026

**Published:** 2026-07-30

**Authors:** Takeru Suzuki, Daisuke Joho, Hiroyuki Okuno, Masaki Kakeyama

**Affiliations:** ^1^Laboratory of Environmental Brain Sciences, Faculty of Human Sciences, Waseda University, Tokorozawa, Saitama 359-1192, Japan; ^2^Department of Biochemistry and Molecular Biology, Graduate School of Medical and Dental Sciences, Kagoshima University, Kagoshima, Kagoshima 890-8544, Japan; ^3^Dementia Pathophysiology Collaboration Unit, RIKEN Center for Brain Science, Wako, Saitama 351-0198, Japan

**Keywords:** circular phase decoding, decision-making, goal-directed behavior, in vivo calcium imaging, mPFC, time-locked activity

## Abstract

Decision-making requires the coordinated integration of sensory information, internal states, and learned rules across time. While the medial prefrontal cortex (mPFC) is known to contribute to this process, how population-level neural activity in the mPFC is temporally organized during decision-making remains unclear. Here, we investigated neuronal population activity in the mPFC of male mice using in vivo calcium imaging while animals performed a touchscreen-based visual cue-dependent location–choice task. We found that mPFC population activity exhibited a stable, low-dimensional, and continuous temporal structure that emerged reproducibly across trials. Using circular phase decoding, we found that these internal population dynamics tracked trial progression across prestimulus, stimulus, and poststimulus epochs. At the single-neuron level, a large proportion of mPFC neurons exhibited reliable, time-locked activity changes around task events. Hierarchical clustering summarized these heterogeneous responses into temporally organized response profiles spanning different phases of the task. Choice-related decoding was observed across these mPFC response profiles, whereas decoding performance in the primary somatosensory cortex remained near chance. Together, these results suggest that mPFC population activity exhibits reproducible temporal organization during visual cue-dependent choice behavior, linking single-neuron heterogeneity with population-level progression dynamics.

## Significance Statement

Understanding how the medial prefrontal cortex (mPFC) contributes to decision-making is a central question in neuroscience. Using calcium imaging in male mice performing a touchscreen-based cue–location task, we found that mPFC neurons exhibited heterogeneous but reproducible temporal activity patterns across trials. Population-level analyses revealed low-dimensional temporal dynamics that tracked progression through the task, whereas hierarchical clustering provided a descriptive summary of temporally organized response profiles. Choice-related decoding was observed across these response profiles. These findings suggest that mPFC activity during choice behavior is organized at both single-neuron and population levels, providing insight into how diverse neuronal responses are coordinated over time during goal-directed behavior.

## Introduction

Decision-making is not an instantaneous event but a process that unfolds over time, integrating sensory information, internal states, and prior knowledge to guide goal-directed actions. During this process, executive circuits must continuously evaluate externally derived information, update internal representations, and prepare appropriate behavioral responses. A large body of work has established that the prefrontal cortex integrates bottom–up and top–down information, including sensory evidence, memory, inference, and planning based on prior experience (Newsome and Paré, 1988; Gold and Shadlen, 2000; Miller and Cohen, 2001; Wimmer and Shohamy, 2012; Ito et al., 2015; Shadlen and Shohamy, 2016; Wassum, 2022).

In line with this integrative view of prefrontal function, the medial prefrontal cortex (mPFC) has been implicated in behavior that depends on previously acquired knowledge. For example, schema-dependent memory studies have shown that rats can acquire structured prior knowledge that supports rapid learning and flexible behavior (Tse et al., 2007, 2011). Such knowledge may provide a framework through which sensory information is interpreted and translated into appropriate choices. These integrative functions may be supported by heterogeneous and mixed-selective neuronal populations in the prefrontal cortex, enabling flexible and context-dependent representations (Rigotti et al., 2013).

Recent studies further suggest that frontal cortical activity can represent temporally extended task structure rather than only isolated stimuli, actions, or outcomes. In rodents, orbitofrontal neurons have been shown to represent future navigational goals during goal-directed navigation (Basu et al., 2021), and PFC neurons can represent abstract behavioral structure across tasks that share a sequence-of-goals structure, supporting transfer to new scenarios (El-Gaby et al., 2024). Related work in humans has also implicated vmPFC in goal commitment and goal-directed attention during an incremental goal-pursuit task (Holton et al., 2024). In addition, encoding of upcoming choices, immediate and final behavioral goals, and multistep action plans has been reported in rodent and primate prefrontal circuits (Saito et al., 2005; Mushiake et al., 2006; Chiang and Wallis, 2018; Sakamoto et al., 2020; Basu et al., 2021; El-Gaby et al., 2024). These findings suggest that frontal cortical populations may organize behavior across extended task epochs by maintaining structured representations of goals, task progression, behavioral context, and upcoming actions.

Despite these advances, how mPFC population activity evolves over time during decision-making that relies on learned cue–choice associations remains poorly understood**.** In particular, it remains unclear whether mPFC population activity exhibits stable and reproducible within-trial temporal organization across repeated trials and how such population-level dynamics relate to the heterogeneous response profiles of individual mPFC neurons. Elucidating these issues is important for understanding how diverse mPFC neurons are coordinated over time during cue-dependent choice behavior.

In this study, we investigated the role of the mPFC in decision-making in mice, with a particular focus on the temporal organization of neural population activity. We used a touchscreen-based operant apparatus (Tamada et al., 2022) and trained mice on a cue–location task in which they learned to associate visual stimuli with specific directional choices (Suzuki et al., 2024) while recording neuronal activity from mPFC neurons using microendoscopic calcium imaging (Ghosh et al., 2011; Chen et al., 2013). We compared mPFC activity with activity in the primary somatosensory cortex (SSp), a sensory cortical control region. To examine population-level temporal organization, we applied Rastermap (Stringer et al., 2025) and phase-decoding analyses. We then used hierarchical clustering to summarize heterogeneous task-related response profiles within the mPFC. Finally, we performed choice-decoding analyses to determine whether and when choice-related information was present in mPFC population activity around the time of action selection.

## Materials and Methods

### Animals

All animal experimental protocols used in this study were performed in accordance with the ARRIVE guidelines (https://arriveguidelines.org) and approved by the Animal Care and Use Committee of Waseda University (A23-098, W23-122). The protocols include justification of the use of animals, their welfare, and the incorporation of the principles of the “3Rs” (replacement, reduction, and refinement), and all efforts were made to minimize the number of animals used and mitigate any potential suffering. Animal breeding and experiments were conducted in the following temperature- and humidity-controlled laboratory conditions: temperature 22 ± 3°C, humidity 40–70%, and 12 h light/dark cycle (lights on: 8 A.M. to 8 P.M.). Five adult male C57BL/6N mice (9 weeks old) were obtained from Charles River Laboratories and housed individually in home cages. Because the food was used as a reward in the behavioral task, feeding was restricted to maintain at least 80% of the average body weight throughout the testing period. Water was provided *ad libitum*.

### Touchscreen operant conditioning apparatus

All animals were tested in operant chambers housed in soundproof boxes. The touchscreen apparatus (O’Hara & Co.) comprised a 15 inch touch-enabled screen, speaker, and food reward dispenser. The touch-sensitive screen consisted of an organic light-emitting diode or liquid-crystal display monitor and a touch-detection unit on one side of the chamber. The trapezoidal chamber (monitor side, 240 mm; reward dispenser side, 55 mm; height, 200 mm) was made of opaque acrylic. The touchscreen was covered with a black plastic plate with three square windows (side dimensions, length, 55 mm; height, 55 mm), spaced 8 mm apart. Visual stimuli were presented on the screen through these windows, and the mice responded by nose poking. Water bottles were installed on both sides of the chamber, allowing the mice to access water *ad libitum* during the task. A food pellet (10 mg each) dispensed from the reward dispenser was used as the reward. A lamp was placed 450 mm from the floor of the chamber to provide illumination during experimental sessions.

### Cue–location task and neural activity recording

The behavioral tasks were initiated after acclimatization of the five male C57BL/6N mice at 17–19 weeks old. The mice underwent viral injection and GRIN lens implantation prior to the task. Acclimatization consisted of two phases, habituation and shaping, each lasting for 2 and 10 d, respectively. During the habituation phase, the mice were familiarized with the apparatus and rewarded with pellets. Two habituation conditions were implemented: Habituation 1 (where the mice could explore the internal chamber and received 10 reward pellets for 15 min) and Habituation 2 (where the apparatus delivered one reward pellet every 30 s for 15 min, accompanied by a sound when dispensing). The habituation phase lasted for a single session of 30 min, comprising both habituation conditions (1 and 2). The following day, the shaping phase began, during which the mice learned that to receive a reward pellet, they had to touch the screen. Each shaping session consisted of 100 trials, with a single trial involving the mice receiving a reward pellet with a sound if they touched the start cue (cross point) in the center window on the screen and lasted for 45 min. If the mouse could not complete 100 trials within the time limit, the session was terminated at 45 min.

**Figure 1.**
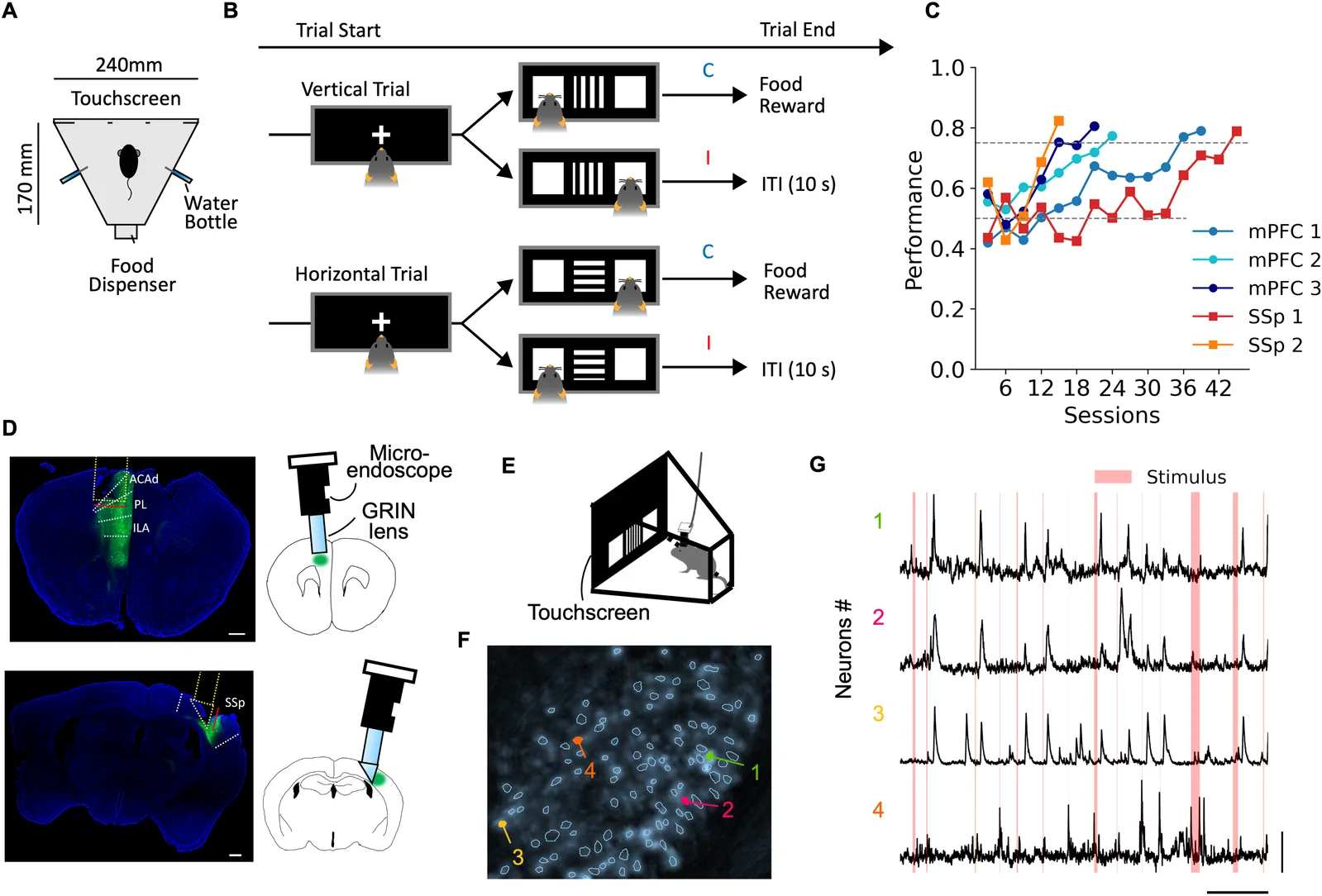
Experimental setup, trial procedure, behavioral performance, and in vivo calcium imaging during the behavioral task. ***A***, A schematic of the mouse in the touchscreen device. ***B***, Procedure of a trial in Task 1 and Task 2. When the mice touched the cross-point cue, a vertical or horizontal stimulus appeared. Mice were rewarded for touching the left window in response to the vertical stimulus and the right window in response to the horizontal stimulus. ***C***, Performance of individual mice. All mice showed progressive improvement in task performance and reached the learning criterion (*n* = 5). Each data point represents the average performance across three consecutive sessions. ***D***, Representative histological confirmation of GCaMP6f viral vector expression and GRIN lens placement in the mPFC (top) and SSp (bottom). mPFC recordings were obtained from the dorsal mPFC, primarily within the anterior cingulate cortex and adjacent prelimbic region. SSp recordings were obtained from the SSp, including the barrel field region. Yellow dashed lines indicate the estimated positions of the lenses, and red dashed lines indicate the estimated focal planes. Scale bars, 500 µm. Green, GCaMP6f; blue, DAPI. ***E***, Schematic diagram of neural activity recording using the touchscreen apparatus and microendoscope. ***F***, Example cell map from one animal. ***G***, Ca^2+^-mediated fluorescence signals from four example cells. Scale bars, 100 s (horizontal) and 5 *z*-score (vertical). C, Correct; I, Incorrect; ITI, intertrial interval; mPFC, medial prefrontal cortex; SSp, primary somatosensory cortex; GRIN, gradient index.

The behavioral task, including correction trials, was conducted over one session per day (Task 1). The start cue first appeared in the central window (Figs. 1*A*,*B*). When the mouse responded, a discriminative stimulus appeared in the central window and two white squares appeared in the left and right windows. The discriminative stimulus was selected pseudorandomly from two possible stimuli (vertical and horizontal). The mice had to touch either the left or right window to obtain a reward according to a predefined rule. The animals learned to respond to the left window when the vertical stimulus was displayed and to the right when the horizontal stimulus was displayed. If the mouse responded to the correct window, a reward was provided, accompanied by a high-pitch tone from the reward dispenser. If the mouse responded to the incorrect window, a low-pitch tone sounded for 1 s, and the lamp was turned off for 10 s as an intertrial interval. All trials began with the appearance of a start cue stimulus in the central window. Correct trials ended by nose poking into the reward dispenser to obtain a pellet, and incorrect trials ended 10 s after responding to the incorrect window. Following an incorrect trial, a correction trial was conducted during which the same discriminative stimulus as that in the incorrect trial was presented. Correction trials continued until the mouse responded correctly and did not count toward the total number of trials completed. The mice were subjected to a maximum of 100 trials (45 min/session). This task was performed until the percentage of correct response rate exceeded 80% for two consecutive times or 75% for three consecutive times. Those meeting this criterion performed the task without correction trials (Task 2) and were given a maximum of 100 trials or 45 min/session for this task. Mice that also met the criteria for Task 2 (>80% two consecutive times or >75% three consecutive times) underwent Ca^2+^ imaging for 20 min to record neuronal activity. Subsequently, the microendoscope (UCLA Miniscope, LabMaker) was removed, and the same task was repeated for 45 min or 100 trials, whichever came first.

### Viral injection and lens implantation

Under general anesthesia, all viruses were injected into the mice at a rate of 100 nl/min, and the needle was removed 10 min after injection to prevent backflow of the virus. For Ca^2+^ imaging, 600–1,000 nl of AAV9-CamKII-GCaMP6f-WPRE-SV40 [Addgene, catalog #100834-AAV9; titer, 2.2 × 10^13^ viral genome copies (gc)/ml] was injected into the right mPFC (*n* = 3; A/P, +1.7 mm; M/L, +0.35 mm; D/V, −1.6 mm from the bregma) or left SSp (*n* = 2; A/P, −1.4 mm; M/L, −3.5 mm; D/V, −0.4 mm from the bregma). At 2 weeks postviral injection, the mPFC mice were implanted with a straight GRIN lens, whereas the SSp mice were implanted with a microprism GRIN lens. For mPFC recordings, the straight GRIN lens (outer diameter, 1 mm) was implanted in the mPFC at A/P, +1.7 mm, and M/L, +0.35 mm, with the ventral surface of the lens positioned at approximately D/V, −1.05 mm from the bregma. Based on the focal distance of the GRIN lens, the estimated imaging plane was located at approximately D/V −1.25 mm from the bregma. Thus, mPFC calcium signals were obtained from the dorsal mPFC, primarily including the anterior cingulate cortex and adjacent prelimbic region. For SSp recordings, the microprism GRIN lens (outer diameter, 1 mm) was implanted at A/P, −1.4 mm (center of the lens); M/L, −2.3 mm for the medial side of the lens; and M/L, −3.3 mm for the lateral side of the lens, with the lateral side of the lens positioned at D/V −1.0 mm from the bregma. SSp recordings were obtained from the SSp, including the barrel field region.

### Ca^2+^ imaging data analysis

Ca^2+^ imaging was performed using a microendoscope at a frequency of 20 Hz. The microendoscope was connected from the inside of the chamber to a digital acquisition device (DAQ) located outside the soundproof box through a flexible coaxial cable. All videos were processed using ImageJ v1.54f and Inscopix Data Processing Software V.1.9.2.3727 (IDPS; Inscopix). First, the raw video data recorded by the microendoscope was converted from 8 to 16 bit using the ImageJ software. The videos were then imported into the IDPS software and downsampled 2× in the *x*- and *y*-directions and spatially filtered (bandpass) with cutoffs set to 0.005 pixel-1 (low) and 0.5 pixel-1 (high), followed by frame-by-frame motion correction. The mean image over the imaging session was computed to calculate the Δ*F*/*F*. Active cells were identified using a combined principal component analysis (PCA) and independent component analysis approach to detect and extract regions of interest (presumed neurons) per field of view (Mukamel et al., 2009). For each video, the extracted output neurons were manually sorted to remove overlapping neurons, neurons with a low signal-to-noise ratio, and neurons with aberrant shapes. The selected neurons and their Ca^2+^ activity traces were further analyzed in Python using custom scripts. Calcium events were detected from *z*-scored Δ*F*/*F* traces by identifying epochs in which the signal exceeded a threshold defined as baseline +2 robust standard deviations, and the peak amplitude within each epoch was used to represent the event.

### Rastermap

Population activity was visualized using Rastermap (Stringer et al., 2025), a dimensionality reduction–based sorting algorithm that orders neurons according to the smoothness and similarity of their temporal activity patterns. For each recording session, Δ*F*/*F* traces were first *z*-scored across time for each neuron and concatenated into a neuron × time matrix. Rastermap was then fitted to this matrix (*n*_PCs = 64; locality = 0.1; time_lag_window = 15), yielding an ordering of neurons that maximizes local temporal correlations while preserving gradual changes across the population. The resulting neuron order was used to display heatmaps of *z*-scored Δ*F*/*F* activity across the entire session and around task events (stimulus onset, choice, and outcome). Rastermap was used solely to obtain a one-dimensional ordering for visualization; all quantitative analyses (e.g., clustering and decoding) were performed on the original, unsorted data.

### Circular phase–based decoding of neural population activity

Calcium Δ*F*/*F* traces were extracted for all recorded neurons, transposed into a neurons × time matrix, and *z*-scored across time for each neuron. The *z*-scored activity was further smoothed with a 1D Gaussian filter (*σ* = 1.0; 10 Hz sampling). For each trial, the interval from trial start to stimulus onset, from stimulus onset to stimulus offset, and from stimulus offset to trial end was treated as three consecutive segments. Median durations of these three segments were computed across trials, and their relative lengths were used to define automatic phase fractions (*r*_1_, *r*_2_, *r*_3_) that partition the unit interval [0,1]. A continuous phase variable ϕ ∈ [0,1] was then assigned along time within each trial, increasing linearly from the beginning to the end of each trial, with segment boundaries given by *r*_1_ and *r*_1_ + *r*_2_. For all valid time points, the smoothed neural activity was reshaped into a samples × neurons matrix and subjected to PCA with whitening. Several candidate numbers of principal components (15, 20, 25, 30) were evaluated, constrained not to exceed the number of samples or features. For each PCA dimensionality, we performed leave-one-trial-out (LOTO) regression using RidgeCV, with groups defined by trial identity. To respect the circular nature of phase, the model predicted sin(2πϕ) and cos(2πϕ), rather than ϕ directly, from the PCA-transformed neural activity. Predicted sine–cosine pairs were normalized and converted back to phase estimates via atan2, yielding circularly consistent predictions $\hat{\varphi }$. Circular mean absolute error (cMAE) between the predicted and true phase was used to quantify decoding performance. The PCA dimensionality yielding the lowest cMAE was selected for subsequent analyses.

### Cell-number–matched and condition–generalization phase decoding

To control for differences in the number of recorded neurons between regions, we performed a cell-number–matched phase decoding analysis. For each mPFC mouse, neurons were randomly subsampled without replacement to match the minimum number of neurons recorded in the SSp datasets (*n* = 81). For each subsampled population, we applied the same PCA-based circular ridge regression procedure used for the main phase decoding analysis. This subsampling procedure was repeated 500 times, and the mean cMAE across repetitions was used as the cell-number–matched decoding estimate for each mouse.

To test whether phase decoding generalized across cue–choice conditions, we performed a leave-one-condition-out analysis using correct trials only. Correct trials were divided into two cue–choice conditions defined by stimulus identity and choice direction; because only correct trials were included, choice direction corresponded to the correct response for each cue. The decoder was trained using all valid time points from one correct cue–choice condition and tested on the other correct cue–choice condition, which differed in both stimulus identity and correct choice direction. The reverse train–test direction was also evaluated. Decoding performance was quantified as cMAE between the predicted and true circular phase. The same analysis was applied to full mPFC populations, cell-number–matched mPFC populations, and full SSp populations.

### Circular-shift null model for phase decoding

To test whether phase decoding could be explained by the temporal autocorrelation of calcium signals, we generated a circular-shift null distribution for each mPFC mouse. For each trial, the neural activity of each neuron was circularly shifted along the time axis by a randomly selected temporal offset. The same offset was applied to all neurons within a trial, preserving the instantaneous population structure and within-trial temporal correlation structure of the calcium signals while disrupting the correspondence between population activity and trial phase. The original trial labels, phase values, and trial boundaries were otherwise kept unchanged.

For each circular-shifted dataset, we applied the same PCA-based circular ridge regression procedure used for the original phase-decoding analysis. This procedure was repeated 500 times per mouse to obtain a null distribution of cMAE. Because lower cMAE indicates better decoding, empirical *p* values were calculated as (*k* + 1)/(*N* + 1), where *k* was the number of circular-shift null samples with cMAE less than or equal to the observed cMAE and *N* was the number of null iterations. With 500 null iterations, the minimum attainable *p* value was 1/501 = 0.002.

### Identification of neurons with significant relevance to decision-making

To determine which neurons significantly responded to decision-making, neuronal activity data (Δ*F*/*F*) from a 9.9 s window (−4.9 to 4.9 s from the visual stimulus onset) for all trials were analyzed (creating two *n* *×* *t* *×* *f* matrices, where *n* is the number of neurons, *t* is the number of trials, and *f* is the number of frames). For each cell, repeated-measures one–way ANOVA (*p* < 0.0001) was used to test whether the neuronal activity was affected by time. Neurons that showed significant changes in fluorescence (Δ*F*/*F*) were classified as “task-related cells,” and those that did not show significant changes in fluorescence (Δ*F*/*F*) were classified as “nontask-related cells.”

### Clustering analysis

Hierarchical cluster analysis was performed to classify neurons based on their trial-averaged Δ*F*/*F* activity. For each neuron, fluorescence signals were *z*-scored within the peristimulus time window (−4.9 to 4.9 s, with stimulus onset defined as 0 s), resulting in a single *n* × f matrix (*n*, number of neurons; *f*, number of time frames). To evaluate clustering solutions with different numbers of clusters, we computed the silhouette coefficient for hierarchical clustering solutions with 2–8 clusters. Silhouette scores were calculated using Euclidean distance on *z*-normalized fluorescence time series. In addition to the silhouette coefficient, the Davies–Bouldin index was computed for hierarchical clustering solutions with 2–8 clusters. The Davies–Bouldin index was calculated using Euclidean distance on *z*-normalized fluorescence time series, with lower values indicating better cluster separation.

To evaluate whether the observed clustering metrics exceeded those expected from a continuously distributed, nonclustered population, we generated unimodal Gaussian null datasets, using an approach conceptually similar to recent analyses of categorical versus noncategorical neural selectivity (Posani et al., 2025). The observed *z*-scored temporal response profiles were first projected into PCA space. The number of principal components was determined by a variance-explained threshold of 85%, which resulted in four components for the present dataset. Surrogate datasets were then generated by independently sampling each principal component from a Gaussian distribution matched to the marginal mean and standard deviation of the corresponding observed PC scores. The same Ward hierarchical clustering procedure was applied to each null dataset, and silhouette scores and Davies–Bouldin indices were computed. This procedure was repeated 1,000 times to obtain null distributions of clustering metrics. Empirical *p* values were calculated as (*r* + 1)/(*N* + 1), where *r* was the number of null samples with a silhouette score greater than or equal to the observed silhouette score, or a Davies–Bouldin index less than or equal to the observed Davies–Bouldin index, and *N* was the number of null iterations.

### Choice decoding from population neural activity

Choice decoding was performed using all recorded neurons for each mouse. Neural activity features were standardized (*z*-scored) across trials at each time point and aligned to the choice time (0 s). A linear classifier was trained to predict left versus right choices at each time point, using neural activity averaged over a ±0.5 s window centered on that time point, with stratified fivefold cross-validation. Balanced accuracy was computed to correct for potential choice biases. To estimate chance-level performance, choice labels were randomly shuffled, and decoding was repeated 100 times to obtain the null distribution. For each region (mPFC, SSp), decoding curves were averaged across mice, and the mean ± SEM was plotted. All analyses were performed using Python (NumPy, Scikit-learn, Matplotlib).

To assess whether decoding accuracy exceeded chance level, we compared the real decoding accuracy to a shuffle-based null distribution at each time point. For each mouse, multiple shuffled decoding time courses were generated by randomizing trial labels. At each permutation, one shuffled repetition was randomly sampled from each mouse, and the mean decoding accuracy across mice was computed. Repeating this procedure 10,000 times yielded a null distribution of mouse-averaged decoding accuracy for each time point. One-sided permutation tests were performed to test whether the real decoding accuracy was greater than the shuffle distribution. Resulting *p* values were corrected for multiple comparisons across time using the Benjamini–Hochberg false discovery rate (FDR) procedure, with a significance threshold of *q* < 0.05.

### Video-based behavioral tracking analysis

To assess possible position- and movement-related confounds in neural choice decoding, we analyzed the behavioral videos recorded during the mPFC imaging sessions. Because the task was performed under dimly lit conditions, the image contrast of the behavioral videos was too low to reliably estimate full-body pose or multiple body-part locations. However, the blue excitation light visible around the miniscope baseplate could be tracked as a single point using DeepLabCut (Mathis et al., 2018). We tracked this blue light rather than the red indicator LED at the top of the miniscope because the baseplate sits closer to the animal's head, providing a better proxy for head position. The *x*-position, *y*-position, and speed of the tracked light were then used as behavioral tracking features, serving as proxies for gross head position and movement.

These features were extracted separately for each analysis window: stimulus onset ±0.5 s, stimulus onset 0–0.5 s, the 1 s period before choice, and choice ±0.5 s. Tracking rate was defined as the fraction of frames within each analysis window in which the head-position proxy signal was successfully detected. For each window, only trials with a tracking rate of at least 0.4 within that window were included. When comparing neural decoding with behavioral-feature-based decoding, the same window-specific trial subset was used for both analyses.

Choice decoding from behavioral tracking features alone was performed using stratified fivefold cross-validation and quantified by balanced accuracy. To evaluate the contribution of position- and movement-related components to neural decoding, behavioral tracking features from the corresponding analysis window were regressed out from neural activity using ridge regression within each cross-validation fold. Choice decoding was then repeated using the residual neural activity. For each decoder, statistical significance was assessed using shuffled-label null distributions (*N* = 1,000). One-sided permutation tests were used to test whether decoding accuracy exceeded the shuffle distribution, because the analysis was designed to assess above-chance decoding. Neural decoding accuracies before and after regression of behavioral tracking features were displayed relative to the 50% chance level.

### Histology

The mice underwent transcranial perfusion with 4% paraformaldehyde in phosphate-buffered saline (PBS). The brains were subsequently removed, postfixed in 4% paraformaldehyde, transferred to a 15% sucrose/PBS solution overnight, and stored in a 30% sucrose/PBS solution at 4°C. Brains were then sectioned into 40 µm coronal sections using a cryostat (Leica) stained with 4′,6-diamidino-2-phenylindole (1:10,000) and mounted on slides. Images were acquired using a fluorescence microscope (KEYENCE BZ-X810).

### Statistical analysis

The rate of correct trials in the tasks excluding correction trials was defined as the correct response rate. MATLAB and Python software were used for statistical analysis. Data are expressed as means ± standard error of the mean unless stated otherwise. Repeated-measures one–way ANOVA (*p* < 0.0001) was used to classify cells as task-related or nontask-related. Hierarchical cluster analysis utilized Ward clustering and Euclidean distance. For time-resolved choice decoding, statistical significance was assessed using shuffled-label null distributions, and *p* values were corrected across time points using the Benjamini–Hochberg FDR procedure (*q* < 0.05). For each cluster, paired *t* test were performed at each time point to compare trial-averaged neural activity between correct and incorrect trials across neurons within the cluster, with Sidak correction for multiple comparisons across time points (*p* < 0.05).

## Results

### Neuronal activity recording during the cue–location task in freely moving mice

Mice were first prepared for in vivo calcium imaging by viral expression of GCaMP6f and implantation of a gradient index (GRIN) lens and were subsequently trained on a visual cue-dependent choice task using a touchscreen apparatus (Fig. 1*A*–*D*), in which choices were guided by visual stimuli and previously acquired knowledge (Suzuki et al., 2024). Each trial began with the presentation of a start cue in the center of three side-by-side windows. Upon touching the start cue, it disappeared, and a visual stimulus (vertical or horizontal stripes) was presented in the same position together with two choice-target panels at both sides (Fig. 1*B*). Mice were rewarded for touching the left panel in response to vertical stripes or the right panel in response to horizontal stripes.

During the training phase, correction trials were introduced such that an incorrect trial was repeated until the correct response was made. In this phase, the correct response rate without correction trials was initially at chance (i.e., 50%) during early training sessions (Fig. 1*C*). Subsequently, all mice gradually increased the percentage of correct responses, eventually exceeding the learning criteria (see Materials and Methods), at which point the association between visual cues and choice directions was considered established. Mice were then trained on the same task without correction trials, and high task performance was maintained after removal of correction trials (Fig. S1*A*).

We prepared and trained three mice for mPFC imaging (Fig. 1*D*) and two for imaging in the SSp as a comparison region. Following successful cue–location task learning (Fig. 1*C*), a microendoscope was attached, and neuronal activity was recorded for 20 min during a task session without correction trials (Fig. 1*E*–*G*). In both regions, all mice exhibited frequent calcium events throughout the task session (Fig. S1*B*–*E*).

### mPFC population activity shows consistent trial-by-trial sequential structure

**Figure 2.**
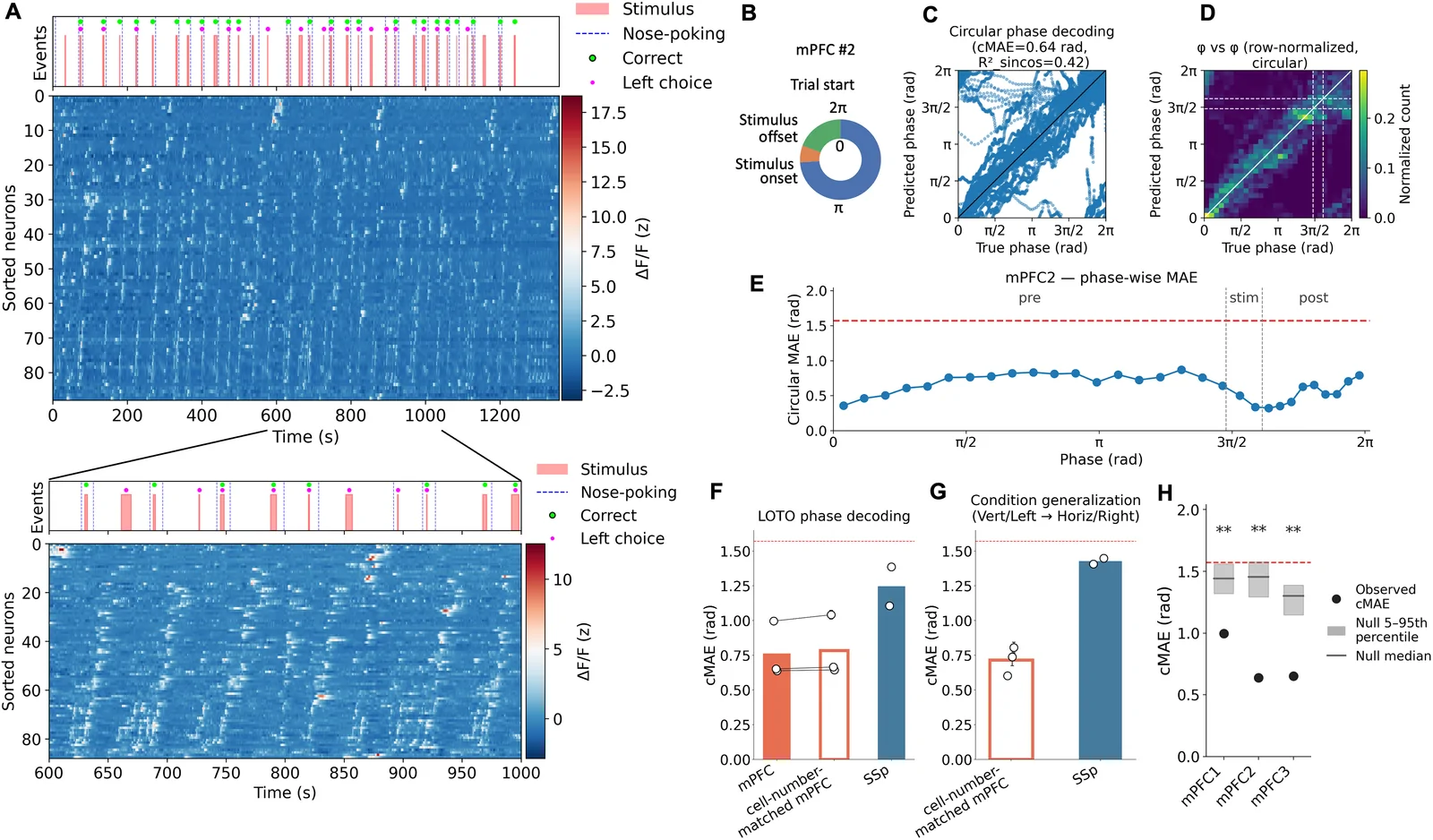
Neural sequence representation and phase decoding. ***A–D***, Example neural sequence and phase-decoding results from mPFC #2. ***A***, Behavioral events, including stimulus onset/offset, nose-pokes, and correct trials, aligned to the continuous neural activity sorted by Rastermap. ***B***, Proportion of decoded phases across the entire recording session for mPFC #2. ***C***, Circular scatterplot of true versus predicted phase (0–2π). The black diagonal indicates perfect prediction. ***D***, Two-dimensional histogram of predicted phase as a function of true phase, row-normalized. Dashed lines mark the automatically identified phase boundaries. ***E***, Phase-wise cMAE across 30 quantile-defined bins. The red dotted line indicates the chance level (π/2). ***F***, LOTO phase decoding with cell-number–matched control. mPFC population activity was decoded using the full recorded population or after randomly subsampling neurons to match the minimum number of neurons recorded in the SSp datasets (*n* = 81). Subsampling was repeated 500 times for each mPFC mouse, and the mean cMAE was used as the cell-number–matched estimate. Lines connect full and cell-number–matched results from the same mPFC mouse. Dots indicate individual mice, and bars indicate mean ± SEM. The red dashed line indicates the chance level (π/2). ***G***, Condition–generalization analysis of phase decoding. The decoder was trained on correct trials from one cue–choice condition and tested on the orthogonal cue–choice condition. For mPFC, neurons were subsampled to match the minimum number of neurons recorded in the SSp datasets (*n* = 81), as in ***F***. cMAE was used as the decoding error metric; lower values indicate better phase decoding. Dots indicate individual mice, and bars indicate mean ± SEM. The red dashed line indicates the chance level (π/2). ***H***, Circular-shift null control for mPFC phase decoding. Black dots indicate observed cMAE values for individual mPFC mice. Gray boxes indicate the 5th–95th percentile range of the circular-shift null distribution generated from 500 iterations, and horizontal gray lines indicate the null median. In each null iteration, neural activity was circularly shifted along the time axis within each trial by a randomly selected temporal offset. The same offset was applied to all neurons within a trial, preserving within-trial temporal autocorrelation and instantaneous population structure while disrupting the alignment between population activity and trial phase. **p < 0.01.

Visualization of neuronal activity using Rastermap (Stringer et al., 2025) revealed a clear sequential pattern in the mPFC that emerged reproducibly across trials (Fig. 2*A*). This observation suggested that mPFC population activity followed a structured temporal progression within each trial. To quantify this organization, we asked whether population activity could encode the instantaneous temporal position within a trial. Because trial progression is continuous, each trial was modeled as a single traversal around a circular phase ranging from 0 to 2π (Fig. 2*B*), and this phase was decoded directly from population activity (Fig. 2*C*–*E*). Circular decoding produced accurate phase estimates in mPFC (cMAE = 0.64 rad; sine–cosine *R*^2^ = 0.42; Fig. 2*C*). The relationship between true and predicted phases exhibited a clear diagonal pattern (Fig. 2*D*), indicating a stable correspondence between neural population dynamics and elapsed trial time. Phase-wise circular error remained consistently below the chance level (π/2 rad) across the entire cycle (Fig. 2*E*), indicating a robust and continuous temporal organization of mPFC population activity.

In contrast, SSp activity showed no consistent sequential pattern across trials in the Rastermap visualization (Fig. S2*A*), and circular decoding performance was markedly poorer in SSp (cMAE = 1.39 rad; sine–cosine *R*^2^ = −0.73; Fig. S2*B*). Consistently, the phase histogram lacked a diagonal pattern (Fig. S2*C*), and phase-wise error frequently reached or exceeded the chance level (Fig. S2*D*).

Across animals, circular decoding errors were consistently lower in the mPFC than in the SSp (Fig. 2*F*), with particularly large differences during the prestimulus and stimulus periods (Fig. 2*G*).

To determine whether the stronger mPFC phase decoding could be explained by the differences in the number of recorded neurons between regions, we repeated the analysis after randomly subsampling mPFC neurons to match the minimum number of neurons recorded in the SSp datasets (Fig. 2*F*). Cell-number–matched mPFC decoding errors remained comparable to those obtained from full mPFC populations and were lower than those obtained in SSp populations, suggesting that the stronger mPFC phase decoding was unlikely to be explained by population size.

We also tested whether the decoded phase signal was preserved across cue–choice conditions (Fig. 2*G*; Fig. S3*A*). Using correct trials only, decoders were trained on one cue–choice condition and tested on the other condition, which differed in both stimulus identity and correct choice direction. Cell-number–matched mPFC populations showed lower cMAE than SSp in both cross-condition analyses, suggesting that the mPFC phase signal was at least partly preserved across cue–choice conditions.

Finally, to test whether phase decoding could be explained by the temporal autocorrelation of calcium signals, we used a circular-shift null model that preserved within-trial temporal structure while disrupting the correspondence between neural activity and trial phase (Fig. 2*H*; Fig. S3*B*–*D*). Observed mPFC decoding errors were lower than the circular-shift null distributions in all three mice, indicating that phase decoding was not explained solely by temporal autocorrelation. These results suggest that the mPFC exhibits a reproducible low-dimensional temporal organization of population activity that tracks trial progression and that this organization was more evident in mPFC than in SSp.

### A larger fraction of mPFC neurons shows task-related modulation compared with SSp

**Figure 3.**
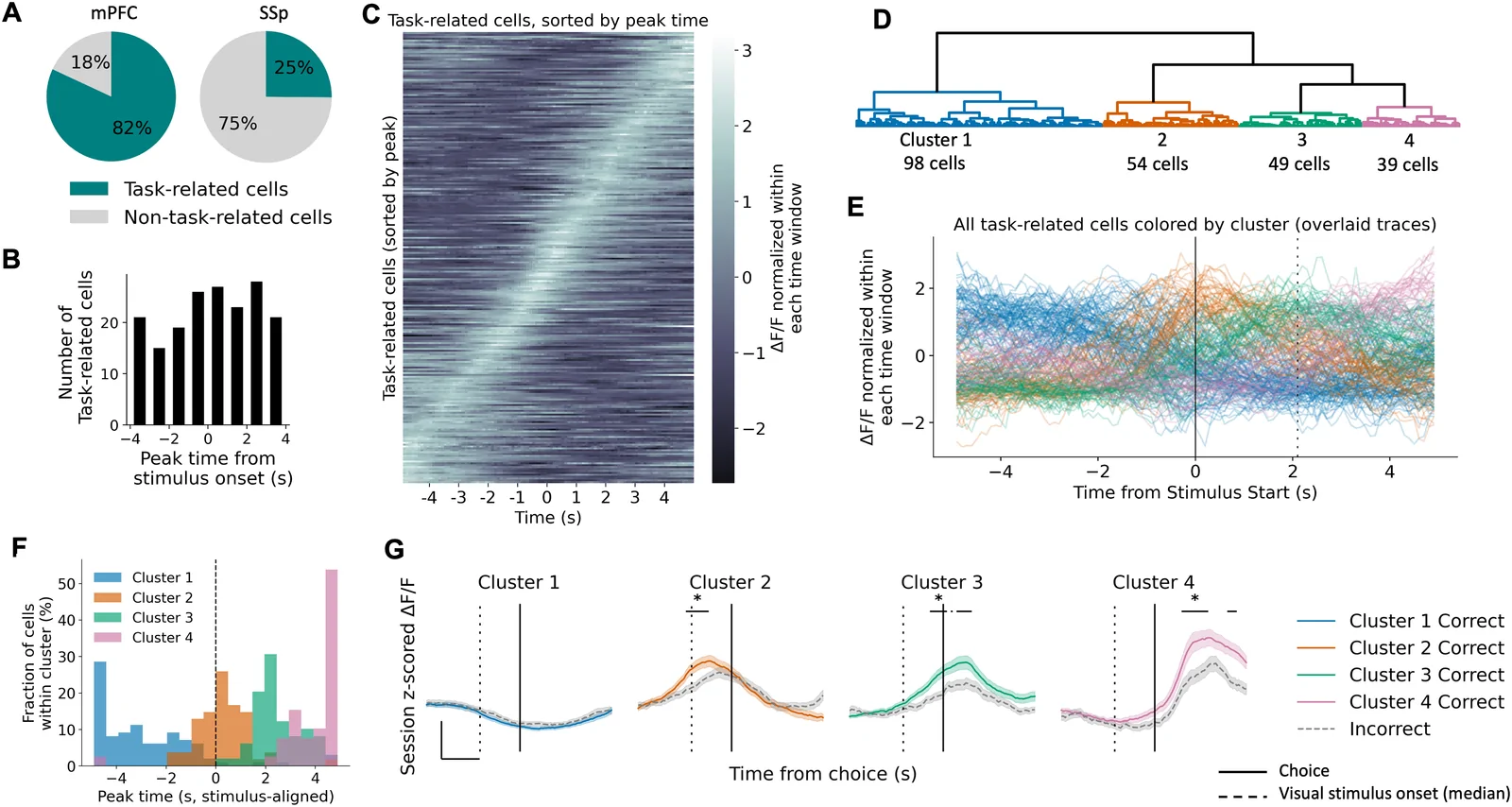
Temporally organized response profiles of task-related mPFC neurons. ***A***, Proportion of task-related cells in the mPFC and SSp. ***B, C***, Task-related mPFC cells have distributed peak timing, as shown by the histogram of the peak timing of window-wise *z*-scored Δ*F*/*F* before and after the visual stimulus onset (***B***) and by the population heatmap (***C***). ***D***, Hierarchical cluster analysis dendrogram and the number of cells in each cluster. Hierarchical cluster analysis was performed using Ward clustering and Euclidean distance based on intertrial mean Δ*F*/*F* data from −4.9 to 4.9 s relative to stimulus onset. ***E***, Session-wise *z*-scored Δ*F*/*F* traces from −4.9 to 4.9 s relative to stimulus onset in each cluster. Vertical dotted lines indicate the median time (2.1 s) relative to stimulus onset. ***F***, Distribution of response peak times for task-related mPFC neurons, colored by the four-cluster assignment. Peak timing was defined as the time point at which each neuron showed its maximum window-wise *z*-scored Δ*F*/*F* response within the stimulus-aligned analysis window. Histograms were normalized within each cluster and are shown as the percentage of cells in each cluster. The dashed vertical line indicates stimulus onset. ***G***, Neural activity traces plotted separately for correct and incorrect trials (mean ± SEM). Vertical solid lines indicate the time of choice. Vertical dotted lines indicate the median stimulus onset time, −2.1 s relative to choice. Scale bars, 2 s (horizontal) and 0.5 *z*-score (vertical). For each cluster, paired *t* tests were performed at each time point across neurons to compare correct- and incorrect-trial activity, with Sidak correction for multiple comparisons across time points (*p* < 0.05). mPFC, medial prefrontal cortex; SSp, primary somatosensory cortex.

To identify neurons whose activity was modulated during the task, repeated-measures one–way ANOVA was applied to Δ*F*/*F* signals within a −4.9 to 4.9 s time window centered on stimulus onset. Neurons exhibiting a significant main effect of time were classified as task-related, while remaining neurons were classified as nonresponsive. Task-related neurons accounted for 65% (190/293 cells) in the mPFC and 25% (50/199 cells) in the SSp (Fig. 3*A*), indicating that a substantially higher proportion of neurons were task-modulated in the mPFC. Moreover, peak activity times of task-related mPFC neurons were broadly distributed before and after the stimulus onset (Fig. 3*B*,*C*), suggesting heterogeneous temporal engagement of individual neurons across task epochs.

### Task-related mPFC neurons show temporally organized response profiles

To further characterize this heterogeneity, we applied hierarchical clustering to task-related mPFC neurons based on their trial-averaged Δ*F*/*F* activity, which was *z*-scored within the peristimulus time window. This analysis yielded a four-cluster solution (Cluster 1, 98 cells; Cluster 2, 54 cells; Cluster 3, 49 cells; and Cluster 4, 39 cells; Fig. 3*D*,*E*). Because clustering algorithms can impose partitions even on continuously organized data, we further evaluated the four-cluster solution using clustering metrics and a unimodal Gaussian null model. The silhouette score did not uniquely favor *k* = 4, whereas the Davies–Bouldin index showed a minimum at *k* = 4 (Fig. S4*A*,*B*). In addition, the observed clustering metrics were better than those expected from the unimodal null distribution (Fig. S4*C*,*D*). However, peak response times were broadly distributed across the trial rather than forming sharply separated groups (Fig. 3*F*). We therefore used the four-cluster solution as a descriptive summary of temporally organized response profiles rather than as evidence for discrete neuronal classes.

**Figure 4.**
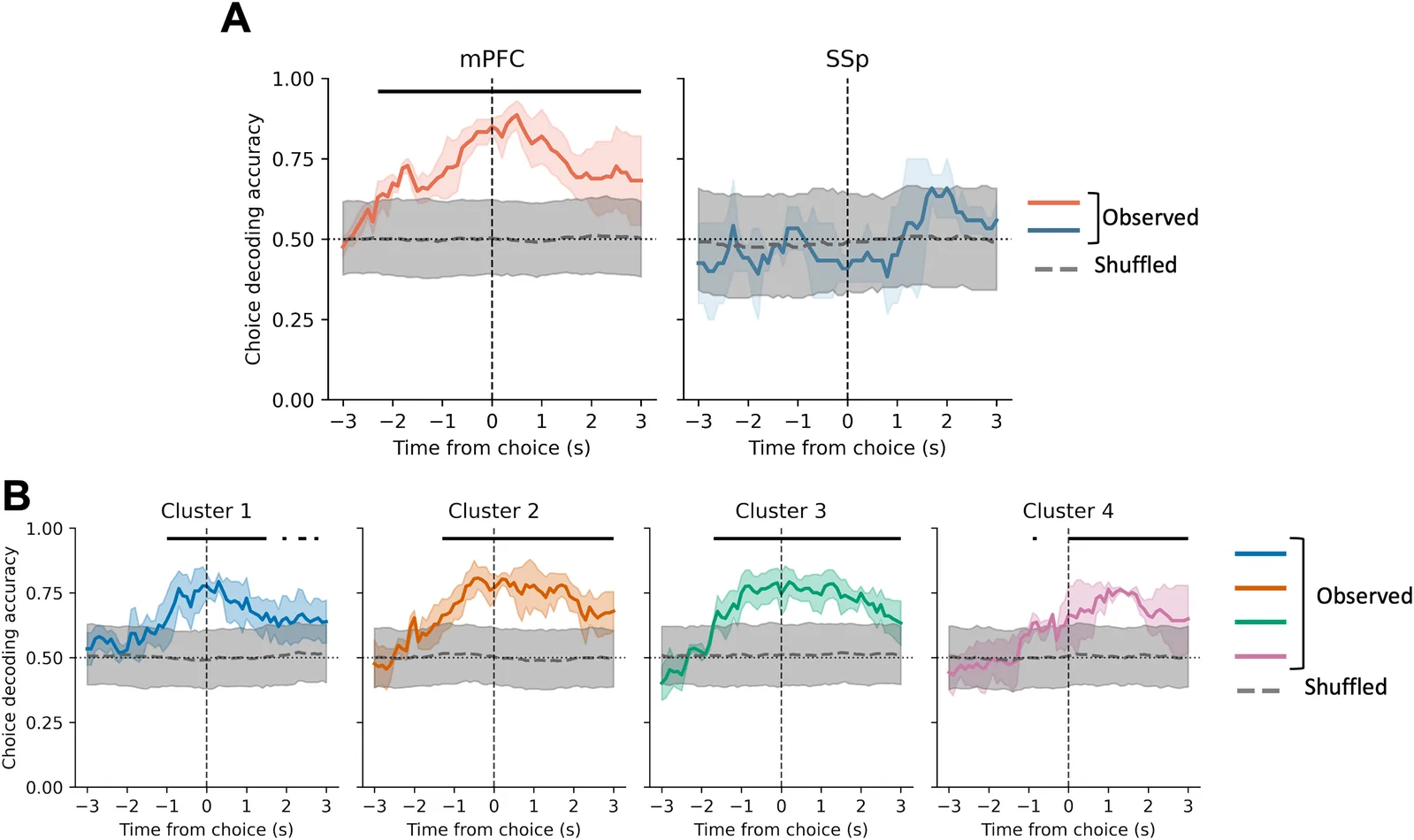
Choice decoding from population activity in mPFC and SSp. ***A***, Time-resolved choice–decoding accuracy for mPFC mice (left) and SSp mice (right), aligned to choice time (0 s). Solid lines indicate mean accuracy across mice, and shaded areas denote ± SEM. Gray dashed lines and shaded areas indicate the median and 5th–95th percentile interval of the label-shuffled null distribution, calculated from bootstrapped group means across mice. Decoding accuracy was compared against the shuffle control using a permutation test across mice at each time point, followed by FDR correction using the Benjamini–Hochberg procedure. Horizontal bars indicate time points with decoding accuracy significantly above the shuffled-label null distribution (FDR-corrected *p* < 0.05). ***B***, Time-resolved choice–decoding accuracy for mPFC neurons grouped by the four temporally organized response profiles shown in Figure 3. The same plotting conventions and statistical procedures as in (***A***) are used.

In mPFC-recorded mice, the median latencies from stimulus onset to choice and reward acquisition were 2.1 and 7.15 s, respectively. Cluster 1 neurons showed reduced activity prior to stimulus onset and relatively low activity around the choice (Fig. 3*G*). Cluster 2 neurons showed transient activation peaking at stimulus onset (Fig. 3*G*; Fig. S4*E*), whereas Cluster 3 neurons exhibited sustained increases following stimulus presentation, with stronger responses in correct than incorrect trials (Fig. 3*G*). Cluster 4 neurons exhibited elevated activity shortly before the choice, with significantly larger peak responses in correct than incorrect trials (Figs. 3*G*, 4*E*). Together, these results indicate that task-related mPFC neurons exhibited heterogeneous temporal response profiles spanning different phases of the task.

### Choice-related decoding is observed in mPFC but not in SSp

Finally, we examined whether population activity could predict the animal's choice direction. Time-resolved decoding of left versus right choices was performed using population activity from all recorded neurons, aligned to the choice time. In the mPFC, decoding accuracy rose significantly above chance prior to choice execution and peaked near the time of choice (Fig. 4*A*; permutation test against chance, *p* < 0.05). In contrast, decoding accuracy in the SSp did not exceed chance at any time point.

Notably, choice decoding from each of the four mPFC clusters was significantly above chance (Fig. 4*B*), indicating that choice-related decoding was observed across temporally distinct response profiles. Together, these results indicate that mPFC population activity, but not SSp activity, was associated with choice direction.

**Figure 5.**
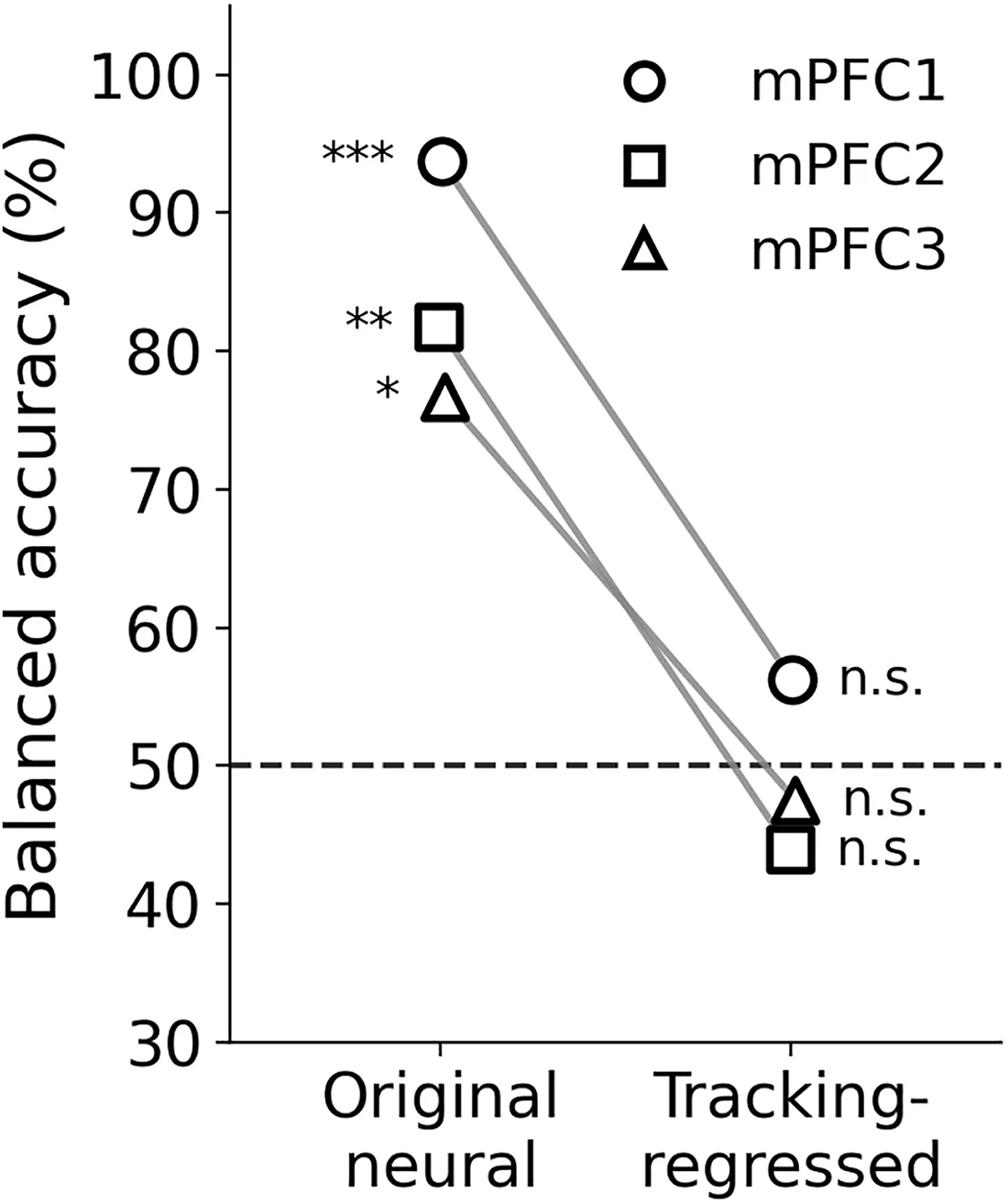
Choice decoding after regression of behavioral tracking features in the choice window. Neural choice decoding was performed using either original neural activity or residual neural activity after regressing out behavioral tracking features extracted from the choice window (choice ±0.5 s). Decoding accuracy was quantified as balanced accuracy using logistic regression with stratified fivefold cross-validation. Dots indicate individual mPFC mice, and lines connect data from the same mouse. The dashed horizontal line indicates the chance level. Statistical significance against chance was assessed using one-sided permutation tests based on 1,000 label shuffles with the same cross-validation procedure. Asterisks indicate significance level (*p < 0.05; **p < 0.01; ***p < 0.001; n.s., not significant).

To assess possible position- and movement-related confounds in choice decoding, we performed an exploratory video-based behavioral tracking analysis using behavioral videos recorded during the mPFC imaging sessions. Because the task was performed under dimly lit conditions, the videos had relatively low contrast, making reliable full-body pose estimation or tracking of multiple body parts difficult across sessions. We therefore tracked the excitation light from the miniscope as a head-position proxy signal using DeepLabCut and used its *x*-position, *y*-position, and speed as behavioral tracking features (Fig. S5*A,B*). These features predicted left versus right choice in all three mPFC mice, indicating that choice direction was associated with measurable differences in gross head position and movement (Fig. S5*C*). We then repeated neural choice decoding after regressing out the corresponding head-position proxy features extracted from each analysis window. After regression, residual neural choice decoding was no longer significantly above chance around stimulus onset in the mouse that showed significant original decoding nor in the prechoice or choice-aligned windows in any of the three mice (Fig. 5; Fig. S5*D*–*G*). These results indicate that choice-related neural decoding in these epochs could not be clearly dissociated from head-position- and movement-related features.

## Discussion

In this study, we investigated how neuronal population activity in the mouse mPFC is temporally organized during a visual cue-dependent choice task. Using a touchscreen-based paradigm, mice selected between two options based on visual stimuli while neuronal activity was recorded from the mPFC and, for comparison, the SSp.

Compared with the SSp, a larger proportion of mPFC neurons exhibited trial-consistent changes in fluorescence around stimulus onset. These stable activity patterns suggest that mPFC activity contains task-related components that are preserved across trials rather than reflecting only transient sensory responses. This stability coexisted with marked heterogeneity at the single-neuron level: some neurons were consistently modulated during stimulus presentation, whereas others showed activity changes before or after stimulus onset. These diverse temporal response profiles suggest that individual mPFC neurons were engaged at different phases of the task.

At the population level, mPFC activity exhibited a sequence-like temporal organization spanning multiple task epochs. Phase decoding further indicated that population activity carried information about the temporal structure of the trial, which may reflect a combination of event-related responses and more continuous trial-progression–related dynamics. These results are consistent with recent studies showing that frontal cortical activity can represent temporally extended task structure, including future goals, task progression, and abstract behavioral structure across trials (Basu et al., 2021; El-Gaby et al., 2024). Our findings extend this literature by showing that mPFC population activity tracks within-trial temporal structure during a touchscreen-based cue–location task and that this temporal organization is more evident in mPFC than in SSp. The condition–generalization analysis further suggests that these trial-progression–related dynamics were at least partly shared across cue–choice conditions.

Hierarchical clustering further grouped neurons with different temporal response profiles. Cluster 2 neurons were activated around stimulus onset and, in some cases, before stimulus presentation, consistent with activity related to preparatory attention or task readiness (Totah et al., 2009). Cluster 3 neurons showed increased activity from stimulus onset until choice execution, which may reflect task-related processes spanning sensory processing, maintenance of learned cue–choice associations, and action preparation. In contrast, Cluster 1 neurons exhibited reduced activity several seconds before choice execution, possibly reflecting suppressive or inhibitory-like activity patterns. Although the underlying circuit mechanisms remain unclear in the present study, previous work suggests that interactions within local prefrontal microcircuits could contribute to such heterogeneous activity patterns (Zhang et al., 2022). Finally, Cluster 4 neurons were activated around choice execution and exhibited stronger responses in correct than in incorrect trials, consistent with activity related to outcome- or reward-related processing. However, because peak response times were broadly distributed and clustering algorithms can impose partitions even on continuously organized data, we interpret these clusters as a descriptive summary of temporally organized response profiles rather than as evidence for discrete neuronal classes. This interpretation is consistent with previous studies emphasizing that apparent neuronal clusters should not be interpreted as discrete functional classes without distinguishing them from continuous or high-dimensional patterns of neural selectivity (Raposo et al., 2014; Posani et al., 2025).

Choice-related decoding was observed across mPFC response profiles rather than being restricted to neurons with a particular response peak timing, whereas SSp decoding remained near chance, suggesting that choice-related signals were broadly distributed across temporally organized mPFC activity patterns. These findings are broadly consistent with prior work showing upcoming choice or action-plan encoding in rodent and primate prefrontal circuits (Saito et al., 2005; Mushiake et al., 2006; Sakamoto et al., 2020; El-Gaby et al., 2024). However, our video-based behavioral tracking analyses indicated that gross head position and movement were associated with choice direction. Head-position proxy features predicted left versus right choice in all three mPFC mice. After regression of these behavioral tracking features, residual neural choice decoding was no longer significantly above chance around stimulus onset in the mouse that showed significant original decoding, nor in the prechoice or choice-aligned windows in any of the three mice. Because left and right choices in the present touchscreen task were directly coupled to distinct orienting and approach movements, these analyses cannot determine whether the decoded neural signals reflected choice-related neural processes, position- and movement-related components, or a combination of both. We therefore interpret these signals conservatively as choice-related activity, without claiming that they represent movement-independent or abstract choice coding. Future task designs that dissociate choice identity from movement direction or body position will be needed to test whether mPFC activity represents abstract choice information independently of action execution.

Together, these findings suggest that mPFC activity during visual cue-dependent choice behavior is characterized by stable, trial-consistent activity at the single-neuron level and reproducible temporal organization at the population level. Although choice-related decoding was observed across temporally organized mPFC response profiles, our behavioral tracking analyses indicate that these signals may include position- and movement-related components. In contrast, phase decoding generalized across cue–choice conditions, suggesting that trial-progression–related population dynamics were at least partly shared across trial types rather than being restricted to a single stimulus, choice direction, or movement trajectory. Thus, mPFC population activity may contain a temporally organized component that reflects progression through the task, alongside choice-period activity that is more closely coupled to action execution.

The heterogeneous temporal activity patterns observed in mPFC neuronal populations could arise, at least in part, from the diverse anatomical and circuit-level organization of the mPFC. The mPFC sends outputs to multiple brain regions, including the amygdala, nucleus accumbens, dorsal striatum, and ventral pallidum (Hoover and Vertes, 2007; Howland et al., 2022), which are implicated in affective processing, motivation, and action selection. In addition, direct and indirect interactions between the mPFC and hippocampal systems have been reported (Varela et al., 2014; Rajasethupathy et al., 2015; Malik et al., 2022; Yadav et al., 2022), as well as top–down influences on sensory processing via thalamic pathways (Nakajima et al., 2019). Such circuit-level diversity may contribute to the temporally organized activation and suppression patterns observed during task performance. However, the present study did not identify projection-defined or cell-type–specific populations. Future work combining calcium imaging with circuit-specific manipulations will be important for determining how distinct anatomical pathways contribute to mPFC trial-progression dynamics.

The choice of SSp as a comparison region should also be considered. We used SSp as a sensory cortical comparison region that was not expected to be directly involved in the visual cue–location association required for this task. This comparison allowed us to test whether reproducible trial-progression dynamics and choice-related decoding were broadly present in a nonprefrontal cortical region under the same behavioral and imaging conditions. However, because the task was visually guided, recordings from visual cortical areas such as V1 would be required to determine whether similar temporal organization is present throughout visual processing stages or is more prominent in higher-order frontal circuits. Thus, the present mPFC–SSp comparison should be interpreted as evidence that these dynamics were more evident in mPFC than in SSp rather than as evidence that they are unique to mPFC among all cortical areas.

In summary, our findings suggest that mPFC population activity during a visual cue-dependent choice task is organized across multiple temporal scales. Individual mPFC neurons exhibited heterogeneous but reproducible task-related activity patterns, while population-level analyses revealed low-dimensional dynamics that tracked progression through the trial. Choice-related decoding was also observed across temporally organized mPFC response profiles, although these signals may include position- and movement-related components. These results place mPFC activity within a broader framework in which frontal cortical populations organize behavior across extended task epochs by maintaining structured temporal dynamics. By integrating a touchscreen-based cue-dependent location–choice task with microendoscopic calcium imaging, this study provides a basis for future work examining how diverse neuronal responses in the mPFC are coordinated over time during goal-directed behavior. Combining projection-specific labeling, cell-type–specific approaches, detailed behavioral tracking, and causal circuit manipulations will be important for determining how temporally organized mPFC dynamics contribute to cue-dependent choice behavior.

## Data Availability

All data are available in the main text or the Supplementary Materials. Requests for the raw data of behavioral task and in vivo imaging should be submitted to kake@waseda.jp.
